# Using comprehensive machine‐learning models to classify complex morphological characters

**DOI:** 10.1002/ece3.7845

**Published:** 2021-06-27

**Authors:** Dequn Teng, Fengyuan Li, Wei Zhang

**Affiliations:** ^1^ State Key Laboratory of Protein and Plant Gene Research School of Life Sciences Peking University Beijing China; ^2^ Peking‐Tsinghua Center for Life Sciences Academy for Advanced Interdisciplinary Studies Peking University Beijing China

**Keywords:** classification, data augmentation, feature extraction, machine learning, morphological characters, SVMorph

## Abstract

1. Recognizing and classifying multiple morphological features, such as patterns, sizes, and textures, can provide a comprehensive understanding of their variability and phenotypic evolution. Yet, quantitatively measuring complex morphological characters remains challenging.
2. We provide a machine learning‐based pipeline (SVMorph) to consider and classify complex morphological characters in multiple organisms that have either small or large datasets.
3. Our pipeline integrates two descriptors, histogram of oriented gradient and local binary pattern, to meet various classification needs. We also optimized feature extraction by adding image data augmentation to improve model generalizability.
4. We tested SVMorph on two real‐world examples to demonstrate that it can be used with small training datasets and limited computational resources. Comparing with multiple CNN‐based methods and traditional techniques, we show that SVMorph is reliable and fast in texture‐based individual classification. Thus, SVMorph can be used to efficiently classify multiple morphological characters in distinct nonmodel organisms.

## Graphical abstract

Recognizing and classifying multiple morphological features can provide a comprehensive understanding of their variability and phenotypic evolution. We provide a machine learning‐based pipeline (SVMorph) including two descriptors and image data augmentation to consider and classify complex morphological characters. We also tested SVMorph on two real‐world examples to demonstrate that it can be used to efficiently classify multiple morphological characters in distinct nonmodel organisms.

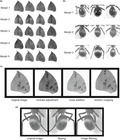

## 1 INTRODUCTION

As intuitive reflections of biodiversity, morphological characteristics provide valuable taxonomic insights into phenotypic evolution and its underlying genetic mechanisms (Grant & Grant, 2006; Joron et al., 2006; Peichel et al., 2001). Therefore, comparative morphology is generally the indispensable, first step of organismal feature identification and investigation. Yet, quantitatively measuring morphological variation remains challenging.

Fortunately, imaging techniques have advanced so that morphological characteristics can be acquired easily. Commonly, morphological features of interest are measured manually and then classified based on measured data, and the usual geometric morphometrics describes individuals through relative landmark positions (Bookstein, 1996). For example, *Drosophila* wings are two‐dimensional structures with clear venation patterns, so the veins and their crossings are usually considered landmarks whose distances and angles can be measured precisely (Cavicchi et al., 1981; Guerra et al., 1997).

However, using those manual methods with large‐scale datasets is both difficult and inefficient. Researchers have developed several advanced methods, such as WINGMACHINE (Houle et al., 2003), DrosoWing (Loh et al., 2017), and FijiWings (Dobens & Dobens, 2013), which detect *Drosophila* landmark information automatically, and MORPHOJ (Klingenberg, 2011), which performs further morphological analyses based on landmark data. Similarly, some automated systems including machine learning‐based methods use fundamental wing morphological features to classify specific insects. For example, using machine learning‐based approaches Crnojević et al. (2014) detected vein junctions to discriminate hoverfly species. Yang et al. (2015) developed DAIIS, which identifies owlfly species according to wing outlines. Also, color pattern modeling characterizes *Heliconius* butterfly phenotypes by comparing color patterns (Le Poul et al., 2014); a machine learning‐based approach classifies guenon face patterns (Allen & Higham, 2015); and Patternize, another machine learning‐based method that works for multiple organisms, uses color pattern variations (Van Belleghem et al., 2018). While those approaches are useful, either for focal organisms or for processing a specific morphological feature, they are not easily used with organisms other than their original targets or for detecting and classifying more complex features that may harbor a wider range of morphological variability in a combination of patterns, shapes, and textures. A comprehensive and efficient solution that considers and handles such integrative features is still required.

As illustrated above, machine learning has recently provided remarkable image processing solutions that can both efficiently identify complex features and accomplish classification tasks (Lürig et al., 2021). Note that although deep‐learning algorithms are widely applied to feature extraction and classification, they require relatively larger training sets and cannot appropriately handle classification problems with fewer samples. In contrast, support vector machine (SVM) universally handles generalized problems and performs well when confronted by a limited or imbalanced sample size, for the decision boundary can be determined by a few support vectors (Tang et al., 2009). Therefore, we developed an SVM‐based pipeline, SVMorph, for complex morphological classification. To begin, SVMorph tackles many classification needs by combining two optional descriptors, histogram of oriented gradient (HOG) (Dalal & Triggs, 2005) and local binary pattern (LBP) (Baraldi & Panniggiani, 1995), to extract morphological features from images, and it then trains an SVM classifier to carry out classification tasks. We also employ data augmentation to improve model generalizability, which aims to increase the dataset size by appropriately modifying existing dataset. To illustrate SVMorph's usage and performance, we used it to classify two groups of organisms that possess distinct and polymorphic features. Ultimately, SVMorph demonstrated its considerable potential and universality by excellently and proficiently handling complex morphological classification.

## 2 SVMORPH WORKFLOW

**FIGURE 1:**
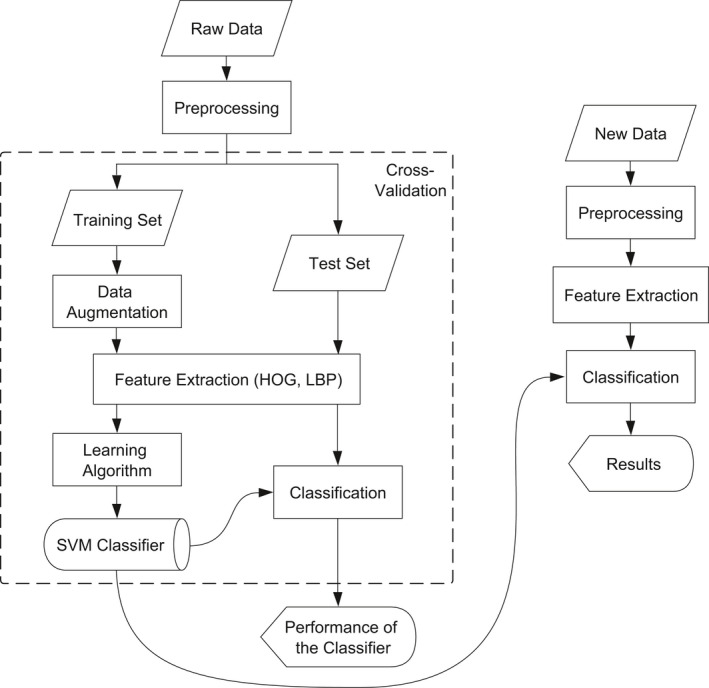
SVMorph framework and workflow. HOG, histogram of oriented gradient (Dalal & Triggs, 2005); LBP, local binary pattern (Baraldi & Panniggiani, 1995); and SVM, support vector machine

We developed SVMorph in the MATLAB environment (MathWorks Inc.). The overall workflow for a typical task generally includes data preprocessing, feature extraction, and classification (Figure 1). When conducting a new task, a classifier must be trained and established before new data may be processed.

## 3 DATA ACQUISITION AND PREPROCESSING

**FIGURE 2:**
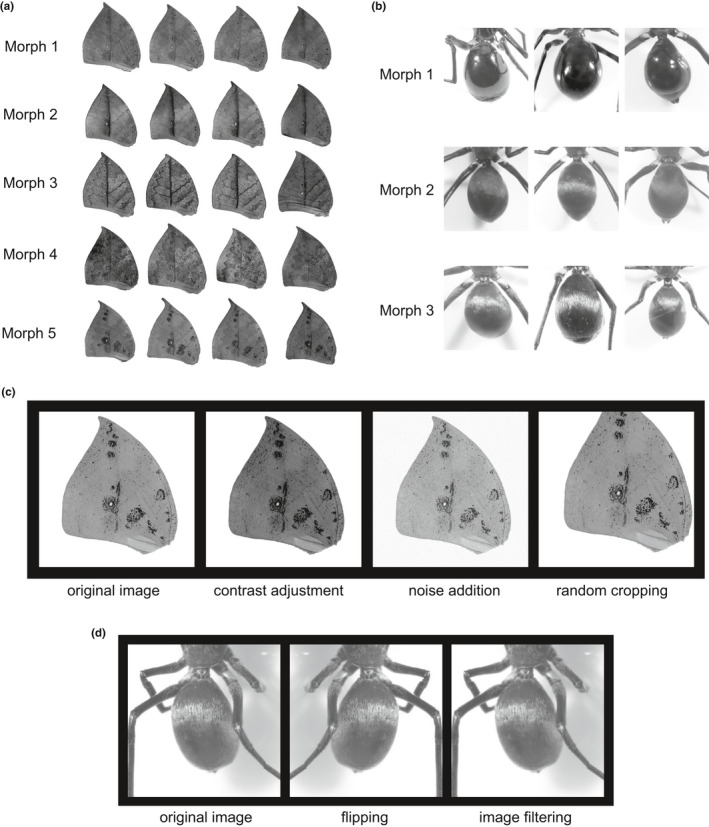
Image data preprocessing and augmentation. (a) Examples of preprocessed grayscale images of polymorphic wing patterns of the dead‐leaf butterfly, *Kallima inachus*. Morph 1: light‐colored main vein; Morph 2: dark‐colored main vein; Morph 3: dark‐colored main vein with lateral veins; Morph 4: light‐colored main vein with dense black dots; and Morph 5: light‐colored main vein with black moldy‐looking spots. (b) Examples of original images showing the polymorphic body textures of the jumping spider species complex in the *Toxeus* genus. Morph 1: mostly hairless abdomen; Morph 2: abdomen with sparse fine black hair; and Morph 3: abdomen densely covered with white hair. (c) Data augmentation examples of contrast adjustment, noise addition, and random cropping of dead‐leaf butterfly wing images. (d) Data augmentation examples showing flipping and image filtering of jumping spider images

While raw images may be captured using digital cameras that can provide better image resolution, we recommend two‐dimensional structures, such as insect wings, be photoscanned for better imaging homogeneity. For example, using a regular scanner (HP LaserJet Pro M227fdw) with a resolution of 600 dpi, we obtained a single forewing image of about 1,000 × 1,000 pixels. While using a digital camera (NIKON D850) with a 1:1 macro lens, we could acquire a single forewing image of over 3,000 × 3,000 pixels with much higher resolution. However, SVMorph or other deep learning‐based algorithms only require 256 × 256‐pixel or 224 × 224‐pixel images as input, and even lower resolutions work as well. Therefore, the need for image resolution is easily satisfied, so we are more concerned with image homogeneity. When the images come from different sources, it may be difficult to ensure the consistency of the photographing equipment or conditions. Relatively, scanners can provide stable photographing conditions and low operational complexity to obtain images with similar quality. Nevertheless, when digital cameras are properly set up, image homogeneity will not be a critical issue. In addition, data augmentation has the potential to reduce the effects of unstable photographing conditions. Therefore, ordinary photographing devices and normal conditions can satisfy the requirements of SVMorph. After data acquisition, SVMorph requires all input image data be preprocessed as follows. Given that both HOG and LBP feature descriptors extract feature information from eight‐bit grayscale images, raw images should be transformed into eight‐bit grayscale format (Figure 2a,b) and the appropriate brightness, contrast, and exposure are required (Figure 2a) to ensure optimum classification accuracy. For our sample datasets, we adjusted the butterfly images with the following parameters: brightness 10 and contrast −50, and we did not adjust the spider images. Note that the adjustments in this step are optional. For photoscanned images, the adjustment in this step is equivalent to setting these parameters correctly when photographing with a digital camera. We want the morphological features we care most about to be well expressed in the images. Therefore, adjustments such as lighting and exposure can depend on the visual effects of the images, that is, whether key morphological features can be clearly distinguished visually. In general, there are no particular restrictions on photographing, but the conditions need to be consistent for a classification task.

Regarding the sample size, as mentioned above, SVM generally performs well compared to other algorithms when faced with a limited or imbalanced sample size, because the decision boundary of SVM is determined only by a few samples (support vectors). Besides, the data augmentation step can improve the classification to some extent as well. Therefore, SVMorph is less affected by the limited sample size. In practice, the criterion of sufficient sample size is difficult to predict and needs to be determined based on the actual model performance. Nevertheless, the model performance generally improves with the increasing sample size, so larger sample size is always preferred. On the other hand, when adding samples, highly imbalanced class distributions of samples should be avoided as well. In practice, we recommend at least 10 samples for each class and the ratio of sample sizes between different classes should be <5. For example, if class A has a sample size of 10 which is the least among all classes, the sample size of other classes should not exceed 50 for each.

## 4 DATA AUGMENTATION

For pattern recognition tasks, a set of preprocessed, classified, and labeled image data functions as a training dataset, and many training samples usually produce the most robust model. However, the practical circumstances of morphological studies underlie the critical need for a data augmentation step in SVMorph, where the rarity of specific specimens often makes sample collection very challenging. Data augmentation artificially creates synthetic training data with label‐preserving transformations by adding slight modifications such as cropping, flipping, rotation, and color jittering, which effectively deal with limited sample size datasets.

**FIGURE 3:**
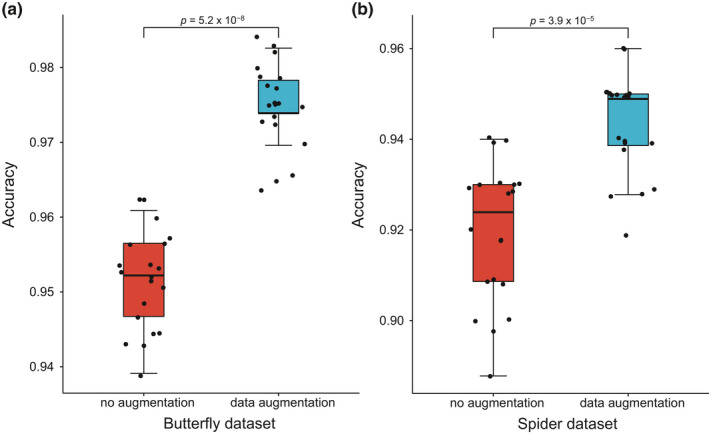
Data augmentation effects on model performance. To compare classification performances before and after data augmentation, we calculated accuracy for each replicate (20 replicates for each group) of 10‐fold cross‐validations for the butterfly (a) and spider (b) datasets, where data are median (*SD*). The average accuracy for the butterfly dataset increases from 0.9513 (0.0064) to 0.9748 (0.0054) after augmentation and from 0.9196 (0.0154) to 0.9433 (0.0110) for the spider dataset after augmentation. Both (a) and (b) show a significantly higher degree of accuracy with augmentation than without augmentation. Wilcoxon rank‐sum test. Boxes enclose scores within the first and third quartiles, and the whiskers show the minimum and maximum values

Additionally, data augmentation may potentially involve both prior knowledge about the data and task‐specific invariances, each of which can regularize the model (Dao et al., 2019). Therefore, to promote task‐specific invariances and generalization of classification models, different augmentation methods are needed for different tasks. Based on data‐specific invariances and features, the preprocessed training datasets may undergo one or more augmentation methods (Figure 2c,d) by implementing the *augmentation.m* script in SVMorph. In our sample datasets, the butterfly wing patterns are less sensitive to parameters such as resolution, brightness, and contrast, so we adopted extensive augmentation methods for butterfly images. As for spider images with texture features more susceptible to the abovementioned image factors, we applied fewer methods to avoid distorting the texture features during the augmentation process. Despite the ubiquity of data augmentation in image classification, its underlying theoretical principles are not well understood. Therefore, in practice, the choice of data augmentation methods often depends on the actual performance. Basically, cropping and flipping methods are more reliable and commonly used, which may be considered as the primary choices. We used random parameters for data augmentation, which refer to the specific input arguments of data augmentation methods. For example, in the noise addition step, we introduced Gaussian white noise with random mean values and random variance values. Thus, different images were augmented differently, which could increase the diversity of the image datasets and avoid introducing bias during the augmentation process. We also tested the effects of data augmentation on the two sample datasets, subsequently demonstrating that data augmentation significantly improved model accuracies in both cases (Figure 3). Therefore, data augmentation is necessary for optimal model performance.

## 5 FEATURE EXTRACTION

**FIGURE 4:**
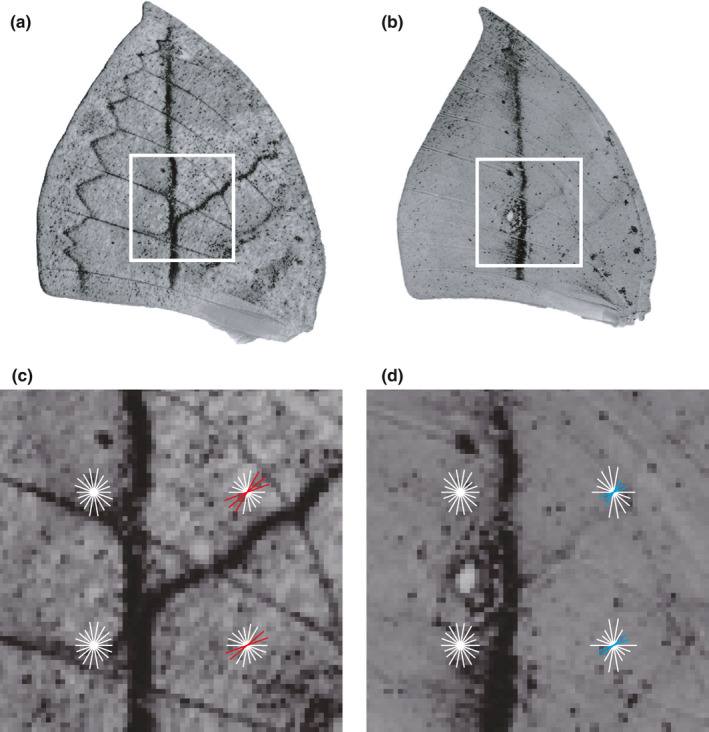
Local histogram of oriented gradient (HOG) feature extractions from the butterfly wing patterns. We chose focal regions (in white boxes) from typical 256 × 256‐pixel image data of *Kallima inachus* Morph 3 (a) and Morph 2 (b), with a cell size of 32 × 32 pixels and a block size of 2 × 2 cells. (c, d) Rose plots of the local HOG features extracted from each specific area in the white boxes in (a) and (b), respectively. Petal length indicates the gradient orientation distribution of a direction within a cell. Petals showing sharp differences between two wing forms are highlighted in red (c) and blue (d)

**FIGURE 5:**
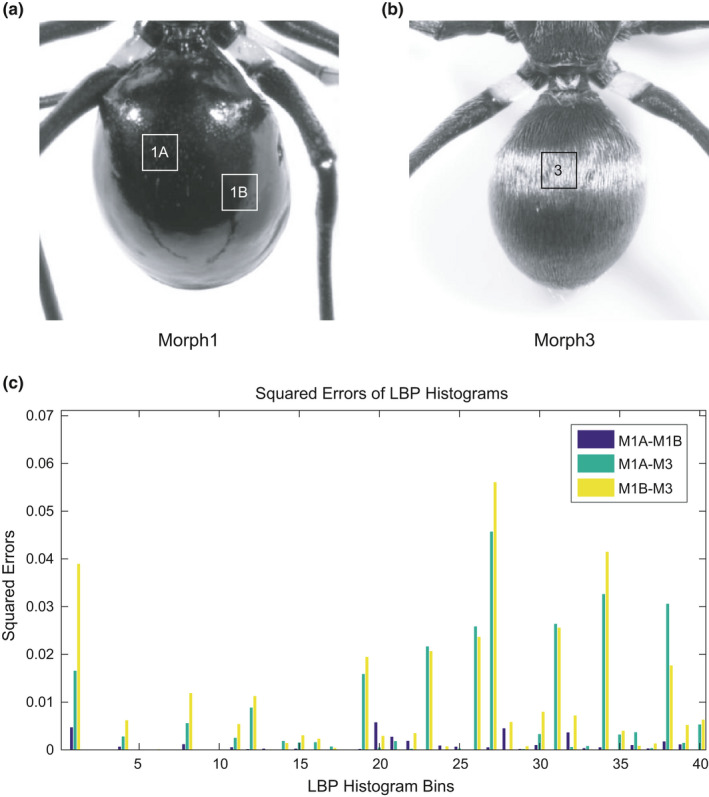
Local binary pattern (LBP) feature extraction of spider body textures. We chose focal regions (in a white or black box) from 512 × 512‐pixel typical image data of *Toxeus* genus Morph 1 (a) and Morph 3 (b), with a cell size of 64 × 64 pixels. (c) Squared errors of the histograms calculated for each cell in boxes 1A and 1B (a) and 3 (b) compare LBP features extracted from the focal regions. Squared errors are relatively smaller for regions with similar textures (M1A–M1B) and relatively larger for regions with different textures (M1A–M3 and M1B–M3)

SVMorph offers two descriptors for feature extraction from the augmented training dataset: HOG (Dalal & Triggs, 2005) and LBP (Baraldi & Panniggiani, 1995). To classify organisms that display a combination of various elements and that produce local edge and gradient information, we recommend HOG as the major feature descriptor. HOG feature extraction is implemented by the *extractHOGFeatures* function in the Computer Vision Toolbox in MATLAB 2020b (https://www.mathworks.com/products/computer‐vision.html). Once implemented, HOG divides images into small spatial regions called *cells*, computes discrete histograms for each cell, and then assembles the cells into larger spatial regions called *blocks* to normalize histograms of all the cells in a block (Dalal & Triggs, 2005), which helps to maintain better invariance and to illuminate changes or shadowing (Figure 4). For texture classification tasks, we recommend LBP be used together with HOG. LBP calculates neighboring pixel gray values to a center pixel and encodes them as a binary number (Baraldi & Panniggiani, 1995), which also endows LBP the advantage of computational simplicity (Figure 5). To further reduce computation time, we use the *extractLBPFeatures* function in the Computer Vision Toolbox of MATLAB 2020b (https://www.mathworks.com/products/computer‐vision.html) to implement a uniform LBP that corresponds to the number of spatial bitwise transitions in LBPs. For example, there would be only 59, instead of 256, total patterns for an eight‐bit LBP operator. Finally, both HOG and LBP (if any) feature vectors from each image are extracted and combined for subsequent downstream classification.

## 6 CLASSIFIER TRAINING

To train multiclass SVM models, we implement the *fitcecoc* function in the Statistics and Machine Learning Toolbox (https://www.mathworks.com/products/statistics.html) in MATLAB 2020b. That function adopts a one‐versus‐one coding design that includes a set of binary SVM classifiers for each possible pair in all classes, so for a model of *n* classes, *n*(*n* – 1)/2 binary SVM classifiers are trained. We improve classification accuracy by applying the error‐correcting output code multiclass model, which is helpful to deal with multiclass classification problems based on multiple binary classifications (Dietterich & Bakiri, 1994). For the classification tasks using example datasets, all feature extraction and classifier training were performed on a computer with an Intel i7‐10700K (3.80G Hz) processor and 32 GB of RAM under a 64‐bit Windows operating system.

## 7 CLASSIFIER TESTING

During the evaluation step, we used *k*‐fold cross‐validation, which partitions all samples into *k* subsets randomly, to examine classifier performances. While a single subset is reserved as test data, the remaining *k*‐1 subsets are used to train the model. A confusion matrix is an *n* × *n* table (for *n* classes), whose rows represent true labels and columns represent predicted labels. The elements on the main diagonal of a confusion matrix indicate correct predictions and, ideally, all off‐diagonal elements equal to zero. The percentage of correct predictions in all test samples defines the classification model's accuracy and, based on the proportion of all main diagonal values in the confusion matrix, is computed as follows:

(1) $$\text{accuracy =}\;\left(\text{TP}+\text{TN}\right)/\left(\text{TP}+\text{TN}+\text{FP}+\text{FN}\right)$$

where TP, TN, FP, and FN stand for true positive, true negative, false positive, and false negative, respectively. To more specifically evaluate a model's performance, we used the precision, recall, and F1 scores that we computed for each class, where

(2) $$\text{precision = TP}/\left(\text{TP}+\text{FP}\right),$$

(3) $$\text{recall = TP}/\left(\text{TP}+\text{FN}\right),$$

(4) $$\text{F1 score}=2\times \text{recall}\times \text{precision}/\left(\text{recall}+\text{precision}\right).$$

**FIGURE 6:**
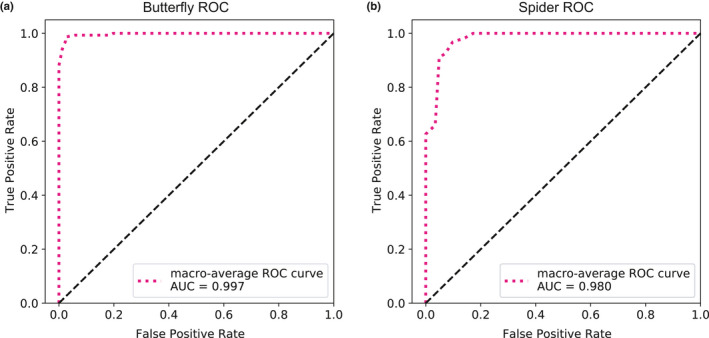
Receiver operating characteristic (ROC) curves for multiclass classifications. Macro‐averaged ROCs and areas under the curve (AUCs) illustrate the predictive abilities of the trained butterfly (a) and spider (b) classifiers. ROC curves plot the true‐positive rate against the false‐positive rate at various threshold settings, and the threshold settings are determined by posterior probability distributions of all the samples. Both ROC curves in (a) and (b) are far from the diagonals, indicating a high level of model performance relative to random guessing

For each of our models, we calculated a confusion matrix for each round of 10‐fold cross‐validations and evaluated the measures calculated from the matrices. We then performed 20 replicates of 10‐fold cross‐validations for each classifier and used the average of 20 results to calculate an estimate of the model's performance. For each model, we created a receiver operating characteristics (ROC) curve by plotting the true‐positive rate against the false‐positive rate at various threshold values. For ROC calculation, all samples were randomly halved into a training dataset and a test dataset and then the training dataset's data were augmented. We used the FitPosterior argument of the *fitcecoc* function to obtain posterior probabilities for the test sample labels. For our multiclass classification model, we first computed ROC scores for each class against the rest of the classes by using the *roc_curve* function in the scikit‐learn toolbox (Pedregosa et al., 2011) in Python 3.9 and then calculated a macro‐averaged ROC as the mean of the binary patterns, which assumes all classes are equally weighted. Area under the curve (AUC) of the macro‐averaged ROC for each model was computed to illustrate their performances (Figure 6).

## 8 OUTPUT

**FIGURE 7:**
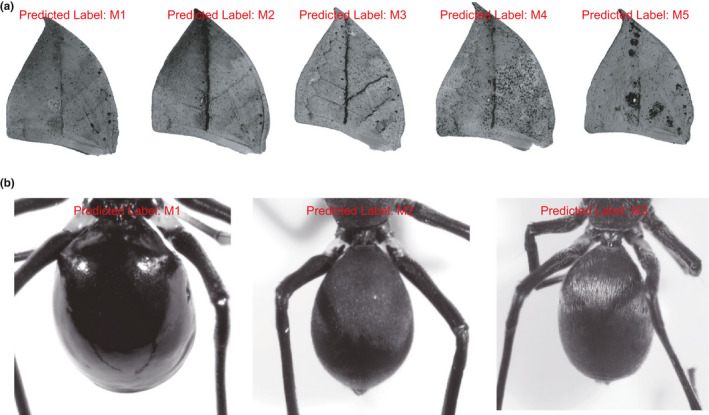
Classifier output that can be used for classification tasks. Examples of the output labels predicted by the trained classifiers for dead‐leaf butterflies (a) and jumping spiders (b) using test data. Labels M1–M5 correspond, respectively, to butterfly morphs 1–5 shown in Figure 2a, and labels M1–M3 correspond, respectively, to spider morphs 1–3 shown in Figure 2b

Our optimally performing, trained and tested classifiers can be used for relevant data classification tasks. Using the *prediction.m* script in SVMorph, we designed classifiers to output predicted labels directly. New data that have undergone preprocessing can be classified using the established classifier, and that results in the most relevant labels being assigned to the image data (Figure 7).

## 9 USAGE EXAMPLES

### 9.1 Wing pattern classification for dead‐leaf butterflies

Dead‐leaf butterflies (*Kallima* spp.) are well known for the leaf patterns on the ventral sides of their wings and are thus a classic example of morphological adaptation (Suzuki et al., 2019). Traditionally, those complex morphological variations have been difficult to quantify precisely and efficiently, so we employed SVMorph to establish an SVM model and to classify those polymorphic leaf wing characters. According to the prior knowledge, we labeled them from Morph 1 to Morph 5 (Figure 2a).

During data acquisition and preprocessing, we photoscanned (HP LaserJet Pro M227fdw) ventral forewing images from 230 K. *inachus* individuals. The subsequent RGB PDF images each had <600 dpi resolution, were extracted using Adobe Photoshop (Adobe Photoshop CC 2018 v19.1.9, Adobe Systems Inc.), and were merged together for batch operations. Then, we preprocessed all the images by first transforming them into eight‐bit grayscale format with brightness and contrast adjusted to 10 and −50, respectively, and then cropped them into separate wing images using the *imagecrop*.*m* script in SVMorph. Then, the images used for the training dataset were classified and labeled manually according to our a priori knowledge (*n*_Morph 1_ = 77, *n*_Morph 2_ = 56, *n*_Morph 3_ = 60, *n*_Morph 4_ = 18 and *n*_Morph 5_ = 19). During data augmentation, preprocessed 256 × 256‐pixel wing images were randomly cropped to 224 × 224 (Figure 2c). Then, we randomly introduced Gaussian white noise with means ranging from 0.1 to 0.2 and variances ranging from 0.02 to 0.05 (Figure 2c). Contrast values used as input limits were randomly adjusted at low intensity values ranging from 0.0 to 0.1 and high intensity values ranging from 0.9 to 1.0 (Figure 2c). Since the butterfly wing patterns appear in a combination of various elements, we selected HOG as the major feature descriptor. In order to capture relative large‐scale wing pattern information while saving computation time, we set 32 × 32‐pixel cells and 2 × 2‐cell blocks for butterfly images of 256 × 256 pixels. We used nine orientation bins that were evenly spaced from 0 to 180 degrees to encode oriented gradient information (Figure 4) and extracted 1,764‐dimensional HOG feature vectors from each image. Considering those high‐dimensional feature vectors, we selected the SVM model with the linear kernel function to reduce computational complexity and overfitting, which also guarantees that the model has fine generalization properties. To evaluate the butterfly classifier's performance, we evaluated accuracy, precision, recall, and F1 scores (Table 1) by using 20 replicates of 10‐fold cross‐validation. Using data augmentation, the butterfly classifier's overall accuracy reached 97.4%, while most classes had fine precision, recall, and F1 scores. However, Morph 5 had a relatively low recall value (86.8%), most likely due to its small sample size or high within‐group variation. The trained model's macro‐averaged ROC curve showed very good predictive ability for butterfly classification (Figure 6a).

**TABLE 1:** Evaluation measures for the butterfly wing classifier (with data augmentation)

	M1	M2	M3	M4	M5	overall
Precision	0.9792	0.9300	1.0000	1.0000	1.0000	0.9818
Recall	0.9760	0.9920	0.9833	1.0000	0.8684	0.9639
F1 score	0.9776	0.9599	0.9916	1.0000	0.9290	0.9716
Accuracy	—	—	—	—	—	0.9748

Note

M1–M5 correspond, respectively, to butterfly morphs 1–5 shown in Figure 2a.

Now using the trained classifier, we classified the preprocessed test data and labeled it accordingly (Figure 7a). The subsequent computational feature extraction and classification processes for this *K. inachus* dataset with 1,764‐dimensional feature vectors took approximately 0.03 s per image to run. According to our results, the butterfly image data were classified and assigned accurately, efficiently, and appropriately to the abovementioned morphs, thus demonstrating the power of SVMorph to extract and distinguish complex wing patterns.

### 9.2 Body texture classification in jumping spiders

*Toxeus* spp. jumping spiders are highly polymorphic mimics that resemble unpalatable *Polyrhachis* ants and thus have a protective advantage (Yamasaki & Ahmad, 2013), providing an excellent example with which to test SVMorph. Among them, members of the species complex of *T. maxillosus*, *T. magna*, and *T. globose* are closely related and have three mimicry morphs according to the body texture features that we named from Morph 1 to Morph 3 (Figure 2b).

Using a microscope (Nikon SMZ18) with an attached digital camera (Nikon DS‐Ri2), we photographed 99 individual spiders. Raw images were captured using SHR Plan Apo 1×, at zoom magnification of 0.75× with Nikon C‐FLED2 LED Light Source. We directly transformed the images into eight‐bit grayscale format and zoomed into the abdominal areas (Figure 2b). Finally, we classified and labeled the images manually according to their phenotype information (*n*_Morph 1_ = 19, *n*_Morph 2_ = 42, and *n*_Morph 3_ = 38). Since the spider data were more sensitive than the previous butterfly wing data, we performed data augmentation in the same way as we did for the butterfly data except for noise addition and contrast adjustment (Figure 2d). The images were filtered using an averaging filter with a hsize of [3 3] and the convolution option. During feature extraction, we used the same HOG parameters that we had used for the butterfly wing images and extracted 8,100‐dimensional HOG feature vectors from each image. In addition, we introduced LBP to extract local texture information. To preserve more local details, we divided the original 512 × 512‐pixel images into 64 × 64‐pixel spatial regions and then calculated uniform LBP histograms for each cell (Figure 5). Specifically, a one‐radius circular pattern was used to compute the LBP of eight neighbors for each pixel, and rotationally invariant features were not considered. Then, L2 normalization was applied to each cell's LBP histogram. As a result, 3,776‐dimensional LBP feature vectors were extracted from each image of the spider dataset, and both HOG and LBP descriptors were combined into 11876‐dimensional feature vectors for spider image classification. As with the butterfly data, we applied the SVM model with the linear kernel function to those high‐dimensional feature vectors. By using 20 replicates of 10‐fold cross‐validations, we calculated accuracy, precision, recall, and F1 scores that helped us evaluate the jumping spider classifier's performance (Table 2). With data augmentation, the overall accuracy of the jumping spider classifier was over 94.3% and, as it was with the butterfly classifier, jumping spider Morph 1, which had the smallest sample size, also had a relatively low recall value (83.4%). These results suggest that as training sample size increases, classification performance should improve. The macro‐averaged ROC curve showed a considerable predictive ability for spider classification, comparable to that of the butterfly classification model performance (Figure 6).

**TABLE 2:** Evaluation measures for the jumping spider classifier (with data augmentation)

	M1	M2	M3	overall
Precision	0.9921	0.9014	0.9734	0.9556
Recall	0.8342	0.9726	0.9645	0.9238
F1 score	0.9051	0.9354	0.9688	0.9365
Accuracy	—	—	—	0.9433

Note

M1–M3 correspond, respectively, to spider morphs 1–3 shown in Figure 2b.

Finally, the preprocessed spider test data were classified using the trained classifier and labeled accordingly (Figure 7b). The feature extraction and classification process for the spider dataset with 11876‐dimensional feature vectors took approximately 0.04 s per image to compute. Our results showed that the spider image data were precisely classified and assigned to the abovementioned morphs, thus suggesting that these spiders could be classified according to their body textures and without any intermediate morph.

### 9.3 Performance of SVMorph and other methods

To evaluate the performance of SVMorph, we compared the accuracy and running time for SVMorph and other methods including the morphology‐based taxonomy and three established deep learning (Convolutional Neural Network, CNN)‐based models such as AlexNet (Krizhevsky et al., 2012), GoogLeNet (Szegedy et al., 2015), and VGG (Simonyan & Zisserman, 2014) (Table S1). All the methods were tested using both butterfly and spider datasets on one Intel i7‐8700 (3.20G Hz) CPU with 16 GB RAM. We also tested three CNN‐based methods on a Quadro P400 GPU, but the VGG‐16 did not complete the task owing to memory overflow, indicating a limitation of GPU usage on such scenarios. For each CNN‐based method, the accuracy was the mean value of ten separate runs to overcome the randomness of CNN model training, whereas SVMorph yielded consistent results. For each method, the time was the mean value of ten separate runs. For the butterfly dataset, we have not established a reliable morphology‐based taxonomy, whereas for the spider dataset, we quantified the spider body textures using a scanning electron microscope (SEM). The SEM images showed that the different body textures were due to different cuticle structures and seta lengths. We collected six individuals for each phenotype and measured three setae for each individual, which took about two days. Relative to the manual morphology‐based taxonomy, all the CNN‐based methods and SVMorph cost less time. However, one of the CNN‐based methods showed relatively lower accuracy in analyzing both datasets. The SVMorph method performed better with considerably high accuracy and the least amount of running time when handling the two datasets, suggesting its application potential on specific and complex classification tasks, and the deep learning‐based algorithms focus on the generalization ability instead of specificity.

## 10 DISCUSSION

The scales and setae of invertebrates are single‐cellular cuticular structures extending from the cuticle, which play an important role in generating phenotypic diversity and can have a profound impact on fitness (Tian et al., 2019). Taken the two sample datasets as an example, the dead‐leaf butterflies in *K. inachus* display a remarkable diversity of leaf wing patterns for masquerade (Protas & Patel, 2008), whereas different morphs of *Toxeus* jumping spiders mimic different ant species to gain a protective advantage (Yamasaki, 2015). These cuticular structures accommodate not only color variation but also shape variation such as length, width, and diameter of a cross section, and pilosity variation (Matsuoka & Monteiro, 2018). Therefore, the complexity in cuticular structures makes it difficult to classify the phenotypes and understand the underlying mechanisms of phenotypic diversity. To characterize the wing coloration of butterflies, one may measure wavelength reflectance in at least three locations: the main vein, lateral veins, and spots. To quantify the texture patterns, one may need to place setae on scanning electron microscopy (SEM) stubs, sputter‐coat them with gold–palladium, and measure their length and width under SEM. However, it is difficult and inefficient to apply these quantifying methods to large‐scale datasets. SVMorph is a convenient and efficient method for identifying variations of cuticular structures such as setae and scales. The method obtained in this study can be used for analyzing wing patterns and body textures, which could fully develop identification systems of phenotypic polymorphism for such nonmodel organisms and bridge the gap between genotype and phenotype. Furthermore, due to the generality of the proposed method, it can be used with no major modification for other tasks, such as analyzing cuticular structures in other invertebrates.

## 11 CONCLUSIONS

SVMorph, a fast and accurate pipeline for handling classification tasks with complex patterns and textures, is particularly helpful for batch processing large image data applications. Also, based on the performance of a trained classifier, it can be used to examine a priori hypotheses of organismal classifications or to extract and investigate images with rare morphological features that cannot be appropriately labeled. Moreover, SVMorph is very modular and easy to schedule, thus giving the researcher the ability to perform each step independently and interactively with other applications. In summary, SVMorph efficiently characterizes and classifies morphological characters of nonmodel organisms.

## CONFLICT OF INTEREST

None declared.

## AUTHOR CONTRIBUTION

**Dequn Teng:** Conceptualization (supporting); Data curation (equal); Formal analysis (lead); Methodology (lead); Resources (lead); Software (lead); Validation (equal); Visualization (lead); Writing‐original draft (equal); Writing‐review & editing (supporting). **Fengyuan Li:** Data curation (equal); Formal analysis (supporting); Funding acquisition (supporting); Resources (supporting); Software (supporting); Validation (equal); Visualization (supporting); Writing‐original draft (supporting); Writing‐review & editing (supporting). **Wei Zhang:** Conceptualization (lead); Data curation (equal); Formal analysis (supporting); Funding acquisition (lead); Investigation (lead); Methodology (equal); Project administration (lead); Resources (equal); Software (supporting); Supervision (lead); Validation (equal); Visualization (equal); Writing‐original draft (lead); Writing‐review & editing (lead).

### OPEN RESEARCH BADGES

This article has been awarded Open Data, Open Materials Badges. All materials and data are publicly accessible via the Open Science Framework at https://github.com/TDQ233/SVMorph.

## Supporting information

Table S1

## Data Availability

SVMorph is available at GitHub (https://github.com/TDQ233/SVMorph) and Dryad (https://doi.org/10.5061/dryad.tdz08kq0g).
